# Physical activity and exercise for the management of chronic low back pain as a component of multimorbidity: from problem to practice

**DOI:** 10.1016/j.bjpt.2026.101621

**Published:** 2026-07-07

**Authors:** Matthew D Jones, Rafael Zambelli Pinto, Manuela L Ferreira, Mitchell T Gibbs

**Affiliations:** a School of Health Sciences, Faculty of Medicine and Health, UNSW Sydney, Australia; b Centre for Pain IMPACT, Neuroscience Research Australia, Sydney, Australia; c Discipline of Physiotherapy, Faculty of Health, University of Technology Sydney, Australia; d Department of Physical Therapy, Universidade Federal de Minas Gerais (UFMG), Belo Horizonte, Brazil; e The George Institute for Global Health, UNSW Sydney, Australia

**Keywords:** Chronic low back pain, Exercise, Multimorbidity

## Abstract

**Background:**

Multimorbidity, characterized by the presence of two or more long-term health conditions, has emerged as a significant health challenge. People with chronic low back pain (CLBP) often live with one or more other long-term health conditions, and this is associated with higher medical costs and worse outcomes. There is a growing need for effective management of multimorbidity in the context of CLBP, but clinicians currently receive limited guidance on how best to do so. Exercise is an evidence-based strategy for the management of both CLBP and associated long-term health conditions, though the way it is prescribed for CLBP differs from that for other chronic diseases. This may limit its effectiveness for managing co-morbidities alongside CLBP.

**Objective:**

The aims of this masterclass are to: 1) describe the prevalence and impact of multimorbidity in the context of CLBP; 2) discuss the role of physical activity and exercise in the management of CLBP as a component of multimorbidity; 3) provide practical recommendations for clinical practice to improve assessment and management of CLBP as a component of multimorbidity; and 4) discuss future research directions to improve management of people with CLBP as a component of multimorbidity.

**Discussion:**

We discuss how clinicians can modify their management of people with CLBP to also address other co-occurring, long-term health conditions through physical activity and exercise. We provide considerations for assessment, as well as examples of how to adjust exercise approaches for people with CLBP who also have additional long-term health conditions.

## Highlights

• We describe the burden of chronic low back pain as a component of multimorbidity.
• We outline the key role of physical activity and exercise for addressing this burden.
• We discuss key considerations for clinicians for managing these individuals.
• We illustrate our proposed approach with a case study.

## Introduction

The global burden of low back pain is well-described. In 2020, low back pain affected 619 million people, with this number projected to increase to over 840 million by 2050.^1^ Low back pain is more prevalent in females and older adults^1^ and continues to be the leading cause of years lived with disability worldwide,^2^ imposing substantial costs.^3^^,^^4^ While most people recover from an acute episode of low back pain, recurrence is common, and many individuals continue to experience ongoing pain and disability for months to years.^5^^,^^6^ Low back pain that persists beyond 3 months is termed chronic low back pain (CLBP) – it is CLBP that contributes most to the global burden.^7^

Alongside the substantial problem of CLBP, multimorbidity has emerged as a significant global health challenge. While there is variation in how multimorbidity is defined, it is often described as the presence of two or more long-term health conditions in an individual.^8^^,^^9^ The global prevalence of multimorbidity is estimated at 37%, though the prevalence appears slightly higher when more conditions are included in defining multimorbidity.^10^ Similar to CLBP, multimorbidity is more common in females and its prevalence increases with age, with more than half the global population over 60 living with two or more long-term health conditions.^10^ Multimorbidity is also more prevalent in people with chronic musculoskeletal pain compared to the general population^11^, ^12^, ^13^ The economic costs and negative effects of multimorbidity on lifespan and healthspan are well established, with greater impacts as the number of health conditions increases.^14^, ^15^, ^16^ There is a clear need for effective strategies to prevent and manage multimorbidity, and to reduce its pervasive impacts.

People with CLBP often live with one or more additional long-term health conditions (referred to hereafter as CLBP as a component of multimorbidity).^11^^,^^12^^,^^17^ Despite this, people with CLBP as a component of multimorbidity are given limited consideration in CLBP clinical guidelines and are often excluded from clinical trials.^7^^,^^18^, ^19^, ^20^ This presents a major challenge for clinicians who may frequently encounter these presentations in practice but are offered little guidance on how to manage these individuals. Therefore, the aims of this Masterclass are to: 1) describe the prevalence and impact of multimorbidity in the context of CLBP; 2) discuss the role of physical activity and exercise in the management of CLBP as a component of multimorbidity; 3) provide practical recommendations for clinical practice to improve assessment and management of CLBP as a component of multimorbidity; and 4) discuss future research directions to improvement management of people with CLBP as a component of multimorbidity.

## Prevalence of chronic low back pain as a component of multimorbidity

**Fig. 1:**
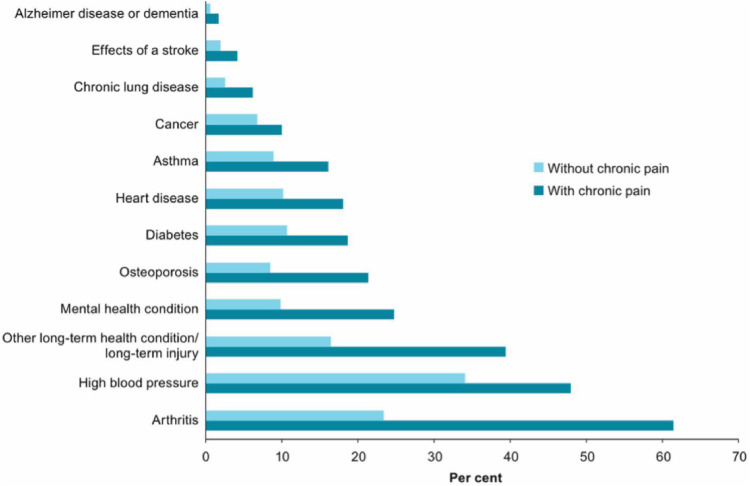
Proportion of people with a long-term health condition, in people with or without chronic pain, aged 45 and over, 2016. Fig. reproduced with permission from Australian Institute of Health and Welfare 2020. “Chronic Pain in Australia”. Cat. no. PHE 267. Canberra: AIHW. License.

Musculoskeletal conditions increase the risk of developing chronic diseases by ∼17%,^21^ so it is unsurprising that many long-term health conditions have a higher prevalence in people with chronic musculoskeletal pain compared to people without pain^22^^,^^23^ (Fig. 1). Most studies in this context have focused on the co-occurrence of CLBP with a single other co-morbidity, such as a cardiovascular, respiratory, metabolic, or mental health condition, and show higher prevalence/greater risk of these conditions in people with CLBP.^24^, ^25^, ^26^, ^27^, ^28^ Studies in high-income countries have also aimed to quantify the co-occurrence of CLBP with a broader range of health conditions, consistently finding higher rates of multimorbidity in people with low back pain than in those without, most commonly osteoarthritis, cardiovascular disease, or mental health disorders.^12^^,^^13^^,^^29^^,^^30^ In low-middle income countries, studies on multimorbidity are somewhat limited, including in the context of CLBP.^31^^,^^32^ A systematic review and meta-analysis of multimorbidity in low-middle income countries found that the prevalence of musculoskeletal pain with other non-communicable diseases was 46%, with hypertension, diabetes, and mental health conditions occurring most often with musculoskeletal pain.^33^ A more recent study of ∼19,000 Brazilian adults with chronic back pain found that the prevalence of multimorbidity was 62%, with cardiovascular disease, arthritis/rheumatism, and depression being the most common co-morbidities.^11^ Taken together, these results show that CLBP as a component of multimorbidity is common in low-, middle-, and high-income countries. In fact, it appears more likely than not that someone presenting with CLBP in practice will have at least one other co-morbidity, particularly if they are older or female. The patterns of CLBP as a component of multimorbidity also appear similar across low-, middle-, and high-income countries.

## Impact of multimorbidity on low back pain

More important than the prevalence of multimorbidity is its associated impacts. For example, compared to those without multimorbidity, the risk of death is 1.73 and 2.72 times higher in older adults with ≥2 or ≥3 morbidities, respectively.^15^ In the context of low back pain, adults with multimorbidity are less likely to receive guideline-concordant care^34^ and the presence of co-morbidities is associated with poorer prognosis for recurrence of low back pain, persistent disability, and progression to CLBP.^35^, ^36^, ^37^, ^38^, ^39^ Key patient-reported outcomes such as pain, disability, physical function, and quality of life are also worse in people with CLBP as a component of multimorbidity compared to those with CLBP alone.^35^^,^^39^, ^40^, ^41^, ^42^, ^43^ Finally, healthcare use and medical costs are higher in people with CLBP as a component of multimorbidity^30^^,^^36^ Together, these findings indicate widespread negative impacts of CLBP as a component of multimorbidity and suggest a clear need for effective interventions to manage both CLBP and co-occurring long-term health conditions.

## Physical activity and exercise for addressing chronic low back pain as a component of multimorbidity

**Fig. 2:**
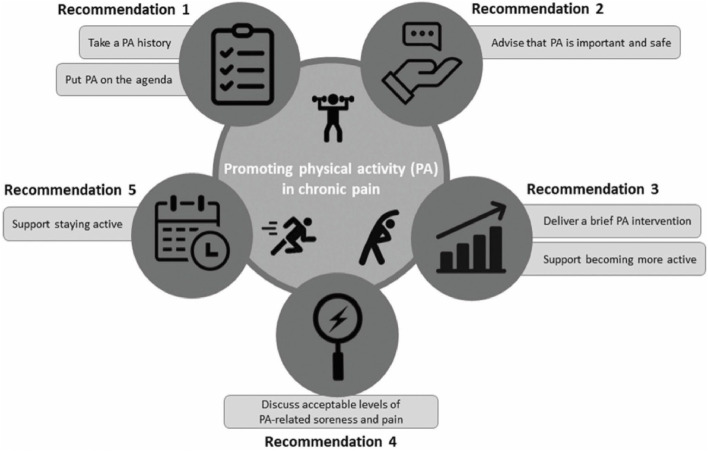
Overview of recommendations to promote PA for individuals living with chronic pain. Fig. reproduced with permission from Vaegter et al. (2024) “Physical activity should be the primary intervention for individuals living with chronic pain A position paper from the European Pain Federation (EFIC) ‘On the Move’ Task Force”, 28(8):1249-1256. License: CC-BY-NC-ND 4.0.

The *Comprehensive Pain Management Editorial Series* published in this journal outlined the growing need for individualized, multimodal lifestyle interventions (e.g., stress management,^44^ sleep,^45^ and physical activity and exercise)^46^ to manage chronic pain.^47^^,^^48^ This Masterclass will focus on physical activity and exercise as the core component of a multimodal lifestyle intervention for people with CLBP as a component of multimorbidity. The European Pain Federation ‘On The Move’ Task Force states that physical activity should be the primary intervention for people with chronic pain and provides five recommendations to promote and support physical activity for these individuals (Fig. 2).^49^ The recent World Health Organization guideline for the non-surgical management of CLBP also recommends exercise as part of a biopsychosocially-informed, multicomponent intervention package.^50^ While this recommendation lacks clear guidance on how to deliver exercise for people with CLBP as a component of multimorbidity, it does consider the values and preferences of people with CLBP and the evidence that exercise is cost-effective, safe, and improves key patient-reported outcomes such as pain, disability, and quality of life.^51^^,^^52^ While research has sought to optimize exercise prescription by examining if there is a best type, dose, or intensity of exercise, the results are mixed and differences in outcomes are often not large enough to be clinically meaningful.^52^, ^53^, ^54^, ^55^ Therefore, it is recommended that exercise be tailored to the person with CLBP's preferences and circumstances, as well as to the knowledge and skills of their treating clinician.^50^ We argue these circumstances should also consider multimorbidity in the context of assessment and management.

Of the various physical, psychological, and pharmacological interventions recommended by clinical guidelines for the management of CLBP,^50^ exercise is arguably the only one that can best address the burden of CLBP in the context of multimorbidity. Indeed, the health benefits of exercise across a range of chronic conditions are well-described, including those that commonly occur with CLBP, such as cardiovascular disease, depression, and arthritis.^56^^,^^57^ However, most of these studies examined the effect of exercise on outcomes in people with a single health condition and did not consider multimorbidity. While exercise appears effective and safe for people with multimorbidity, this is based on low-certainty evidence from a small number of trials across a narrow range of multimorbid presentations, none of which included CLBP.^58^ However, more recent clinical trial evidence supports the role of lifestyle management in people with CLBP and co-existing medical conditions^59^ as well as in people with chronic musculoskeletal pain in the context of multimorbidity.^60^ More trials of co-morbidity adapted exercise interventions in people with CLBP are currently underway.^61^ We note that other study designs apart from tightly controlled randomized trials focused on efficacy could also be used to inform this evidence base. This might include more pragmatic randomized trial designs that better assess the real-world effectiveness of interventions, or observational designs to investigate common phenotypes and prognosis in people with CLBP as part of multimorbidity. Emerging methods, such as target trial emulation, could also be used to investigate the effectiveness of interventions while reducing design- and analysis-related biases that limit the applicability of some observational research.

## Influence of physical activity, exercise dose and adherence

The argument that physical activity and exercise are best placed to simultaneously improve CLBP and co-occurring long-term health conditions is based on two key tenets: 1) that exercise is adequately dosed, and 2) that exercise is being adhered to. However, this relationship is not straightforward, as the required dose and adherence to exercise for improving pain and disability may differ from those needed for the prevention and management of chronic disease. Declining exercise adherence has been proposed as a reason for the diminishing effects of exercise over time, though there is currently limited direct evidence to support this. While high exercise adherence (>80%) is associated with the largest improvements in pain (0-100 scale: mean difference (MD) [95% confidence interval (CI)]: -14.3 [-18.6 to -10.0]) and disability (0-100 scale: MD [95%CI]: -8.1 [-10.7 to -5.5]) in people with CLBP, the differences compared to moderate (60-79%) and low adherence (<59%) are small and not clinically meaningful (pain: high v low adherence (-8.9 [-15.3 to -2.6]), moderate v low adherence (0.8 [-5.9 to 7.5]; disability: high v low adherence (-3.6 [-7.4 to 0.1], moderate v low adherence (1.7 [-2.5 to 5.9]).^62^ Thus, strict adherence to physical activity and exercise may matter less for improving pain and disability in people with CLBP, though it may reduce the odds of developing CLBP.^63^ In contrast, associations between physical activity and exercise with improvements in other chronic disease outcomes (e.g., morbidity and mortality) are more apparent, so adherence may matter more in this regard.^64^^,^^65^

**Fig. 3:**
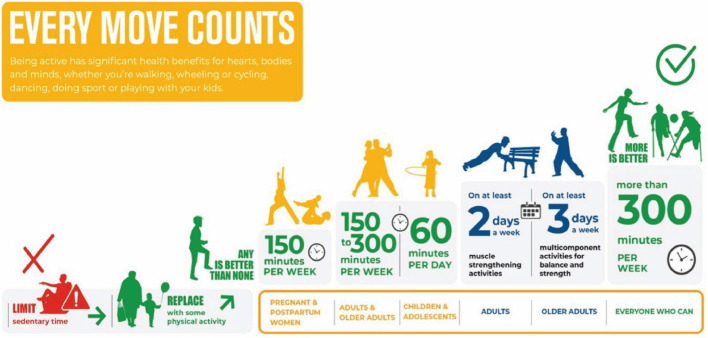
Summary of physical activity recommendations by the World Health Organization. Fig. reproduced with permission from “WHO guidelines on physical activity and sedentary behaviour: at a glance” Geneva: World Health Organization; 2020. Licence: CC BY-NC-SA 3.0 IGO.

For general health benefits, the World Health Organization recommends adults accumulate at least 150–300 min of moderate-intensity aerobic physical activity, or at least 75–150 min of vigorous-intensity aerobic physical activity, or an equivalent combination of the two, as well as twice-weekly moderate-intensity muscle-strengthening exercise targeting major muscle groups.^66^ The physical activity guidelines also recommend reducing sedentary time and acknowledge that doing some physical activity is better than none (‘Every Move Counts’), as this still confers health benefits (Fig. 3).^66^^,^^67^ In support of this, improvements in health are apparent with ‘exercise snacks’ and moderate-to-vigorous intermittent lifestyle physical activity, despite small total amounts of weekly physical activity.^68^^,^^69^ For CLBP, the dose of exercise appears to have a limited effect on improving pain and disability.^53^ As such, having people with CLBP working towards or meeting the physical activity guidelines may result in similar pain and disability outcomes to a low volume/intensity program (e.g., motor control, core stabilization),^50^^,^^70^ but with the added benefit of positively impacting the prevention and management of multimorbidity. However, very few exercise programs tested in clinical trials of people with CLBP are adequately dosed to meet these guidelines.^71^ Moreover, improvements in physical factors by exercise appear less related to clinical outcomes than psychological factors (e.g., reduced fear avoidance and catastrophizing), which are more consistent mediators of improved pain and disability.^72^, ^73^, ^74^ These psychological mediators may be less influenced by exercise volume and intensity than those for other chronic conditions (e.g., inflammation, cholesterol, and insulin sensitivity for cardiometabolic diseases).^75^, ^76^, ^77^ Therefore, while back-specific, low volume/intensity exercise can improve pain and disability in people with CLBP, we argue that a more holistic physical activity approach should be adopted to confer additional health benefits in the context of multimorbidity (where relevant for the individual) while maintaining the same potential for clinical improvements in pain and disability. In practice, this would emphasize not only increasing structured exercise but also strategies to reduce sedentary time and increase incidental physical activity, with the goal of sustaining increased physical activity in the long term.

## Improving adherence to physical activity and exercise in people with CLBP as a component of multimorbidity

Despite the health benefits of physical activity, 30% of adults do not meet the aerobic activity guidelines, and less than 20% meet both the aerobic and muscle-strengthening recommendations.^78^^,^^79^ Pain and functional limitations from CLBP, as well as negative signs and symptoms associated with co-morbid chronic disease, can make regular physical activity difficult, which may explain the sometimes low levels of physical activity in these populations.^80^, ^81^, ^82^ Moreover, the behaviour of physical activity is complex, and helping people become more physically active is challenging; even those who want to increase their activity often fail to do so.^83^ People with CLBP experience multiple barriers to exercise, including co-morbidities, that differ within- and between-individuals and can change relative to the timepoint of exercise (i.e., before, during, or after exercise).^84^ Thus, strategies aimed at improving physical activity and exercise in people with CLBP as a component of multimorbidity must account for this dynamic complexity.

Previous studies have attempted to determine the effectiveness of interventions for increasing physical activity in people with chronic pain, as well as the intervention components and behaviour change techniques that may be most important for doing so. Current interventions investigated in people with chronic musculoskeletal pain have only a small, short-term effect on increasing physical activity.^85^^,^^86^ Common components of interventions shown to increase physical activity include the use of a combined physical activity and behaviour change approach that is tailored to the individual and is delivered by a health professional.^87^ Behaviour change techniques, including education from a credible source, outcome monitoring, feedback, and goal setting, are common among effective interventions and may assist with adherence.^85^^,^^88^ Clinical guidelines recommend that goal setting be person-centered, where the individual’s needs and values guide the formulation of goals and the strategies to work towards them. This should involve a collaborative approach with the clinician and may lead to greater treatment engagement and better outcomes than clinician-led goal setting.^89^

## Key considerations for clinicians

From a practical standpoint, several key considerations for clinicians can help individuals with CLBP, as part of multimorbidity, become more physically active. The first is health education. People with CLBP often have physical activity and function-related goals,^90^^,^^91^ but many are not aware of the physical activity guidelines and why they exist.^92^ Thus, a useful first step for some may be education around physical activity and how this might help the person return to their meaningful activities. This should include assessment of physical activity levels and advice on the amount of physical activity recommended for health benefits, as well as the positive role of physical activity for reducing pain, improving function, and managing associated co-morbidities. If people with CLBP are worried that increasing their physical activity will worsen their pain, clinicians should explore these concerns and beliefs and provide tailored reassurance. This could include advice that exercising with some pain is safe and that any increases in pain with exercise are transient and do not lead to worse outcomes.^93^^,^^94^ People with CLBP as a component of multimorbidity should also be reminded that even small amounts of physical activity, be it incidental or planned, are associated with substantial health benefits, even if physical activity levels remain below those recommended by guidelines.^68^^,^^95^

A second important consideration is kinesiophobia, defined as a specific fear that movement or activity is believed to cause injury or reinjury.^96^ Kinesiophobia is a prevalent phenomenon across a variety of chronic conditions (e.g., cardiovascular disease, respiratory disorders, and chronic pain) that can be a significant barrier to physical activity.^97^ Identifying and addressing kinesiophobia in people with CLBP as a component of multimorbidity may be a necessary step for helping them to become more physically active. Strategies for how to do this in the context of pain have been described in detail elsewhere^98^ and similar strategies would likely be effective for reducing kinesiophobia related to other chronic conditions as well (e.g., cardiac anxiety).

Another consideration is the person’s beliefs and expectations. People with CLBP often want a specific diagnosis and treatment,^99^, ^100^, ^101^ with back-focused specific exercise typically considered more beneficial than general exercise (e.g., aerobic and whole-body resistance exercise) despite a lack of difference in outcomes between the two approaches.^102^ People with CLBP also value exercise that is tailored to them, though this does not have to be specific exercise. Indeed, previous studies have shown people with CLBP perceive general exercise as individualized to them, particularly if they are told it will help with their pain, resulting in similar adherence and outcomes to specific exercise.^103^^,^^104^ Therefore, while people with CLBP may prefer specific exercise, education about the efficacy of general approaches appears to negate any potential negative impact on adherence and outcomes. This education should also include discussion of the increased ability of general exercise to manage multimorbidity through a more holistic physical activity approach.

The final important consideration is the clinical context in which care is delivered. The expectations of people with pain are associated with outcomes^105^^,^^106^ and are likely an important contributor to the contextual effects of interventions. The quality and strength of the patient-practitioner relationship (e.g., trust, empathy, therapeutic alliance) are also linked to improved outcomes and better exercise adherence in people with chronic pain.^107^^,^^108^ While the effectiveness of interventions designed to enhance these contextual effects on pain and disability in people with chronic pain is uncertain,^109^ we nonetheless remind clinicians of the power of the therapeutic clinical encounter, including their role in shaping patient expectations and fostering a strong therapeutic alliance, to potentially improve outcomes.

## Case study

Table 1.

**Table 1:** Case study example of the management of a person with chronic low back pain as a component of multimorbidity.

Interview
*History:* A 53-year-old male lawyer who has experienced persistent low back pain for the past 5 years. The pain began after lifting a 20kg suitcase while returning from a family holiday with his wife and two teenage children. At the time, he felt a jarring sensation in his lower back but managed to complete the journey home. The pain gradually worsened over the following days and has persisted on most days since. *Pain characteristics:* Described as persistent, dull, and aching, sometimes radiating in a poorly localized pattern to the upper buttock and posterior thigh. Denies any sharp, shooting pain, numbness, tingling, or leg weakness. Pain is aggravated by prolonged sitting, certain postures, and bending or lifting. *Other medical history:* Family history of cardiovascular disease (father and grandfather have a history of heart attack). Diagnosed with Stage 1 hypertension by his GP ∼2 years ago, which is now controlled through antihypertensive medication. Had previously been managing low back pain with intermittent use of NSAIDs but was advised to discontinue these by the GP, who explained that these drugs could reduce the effectiveness of his antihypertensive medication and increase cardiovascular risk. Now relies on paracetamol for pain relief as needed. *Lifestyle factors:* Sedentary (including prolonged sitting at work, up to 8h/day) and physically inactive. Only reports occasional walking during the week, but no other structured activity. Reports daytime fatigue that worsens in the afternoon. Has a BMI of 30.1 (obese). Occasionally wakes feeling unrefreshed, otherwise does not report problems with sleep. *Thoughts and beliefs*: Avoids physical activity out of fear that exercise causes further damage to the lower back and worsens pain. Has grown increasingly concerned about the long-term ability to work and is frustrated with the ongoing impact of pain on both personal and professional life. Growing stress and anxiety around cardiovascular health, particularly given a hypertension diagnosis and a family history of CVD. Management plan considerations *Overview:* Adopt recommendations of clinical guidelines for CLBP (clinical assessment with referral as appropriate, personalized information and advice, multimodal care based on shared decision-making). Adopt recommendations of clinical guidelines for hypertension (pharmacological management and lifestyle modification, with emphasis on physical activity (aerobic and resistance exercise)). *Additional considerations:* *Assessment:* Conduct pre-exercise screening to determine suitability for physical activity (e.g., ACSM or ESSA tools). Assess sedentary behaviour and physical activity levels (e.g., IPAQ or PAVS questionnaires). *Education:* Pain science education (e.g., hurt does not equal harm, pain with exercise, pain and hypertension). Role of lifestyle modification (stress, diet, etc.) for management of CLBP and hypertension Physical activity guidelines and importance of small increases in physical activity (‘Every Move Counts’) Role of physical activity for addressing CLBP, hypertension, and CVD risk. *Intervention:* Pain education and graded exposure to address kinesiophobia and problems with bending and lifting Collaboratively develop strategies to reduce sedentary time at work Collaboratively develop strategies to increase incidental and structured physical activity Behaviour changes techniques to support long-term engagement in physical activity

Abbreviations: ACSM, American College of Sports Medicine; BMI, body mass index; BP, blood pressure; CLBP, chronic low back pain; CVD, cardiovascular disease; ESSA, Exercise and Sports Science Australia; GP, general practitioner; IPAQ, International Physical Activity Questionnaire; NSAIDs, non-steroidal anti-inflammatory drugs; PAVS, Physical Activity Vital Sign;

## Potential barriers to implementation and future directions

The barriers faced by clinicians when trying to implement biopsychosocial, evidence-based care for people with musculoskeletal pain are well described.^110^^,^^111^ In contrast, relatively few studies have investigated these barriers in the context of chronic pain as a component of multimorbidity. The limited evidence shows most physical therapists believe they should adapt their approach for people with chronic pain as a component of multimorbidity, but are challenged by a lack of time (which may lead to sub-optimal history taking and pre-exercise screening) and the perception that people with multimorbidity may be less able to self-manage.^112^ There are also challenges around the scope of practice, with disagreement about which conditions should be treated by the physical therapist versus other health professionals (e.g., the person’s doctor or psychologist), as well as who is responsible for coordinating the person’s care.^112^ These issues led physical therapists to perceive they could not achieve the same clinical improvement for people with chronic pain as a component of multimorbidity in the same timeframe. Interestingly, the values and expectations of people with musculoskeletal conditions as part of multimorbidity towards their physical therapists appear less tied to treatment outcomes and more centered on building strong relationships and trust, as well as on sharing power and responsibility for their own care.^113^ This aligns with qualitative research on general practitioners, where building and maintaining strong long-term relationships are the key aspect of their care for people with multimorbidity.^114^

There are a few areas of future research that could help improve the management of people with CLBP as a component of multimorbidity. First, there is a need for more studies examining the effectiveness and safety of physical activity and exercise-based approaches in people with CLBP as part of multimorbidity, and how these can be optimized. These studies might then inform future clinical guidelines, allowing them to provide more specific recommendations for management of those with co-morbidities. Second, future qualitative research should explore the perspectives of clinicians and people with CLBP as part of multimorbidity to better understand their expectations regarding management and clinical responsibility, as well as the role of health systems in facilitating this. Together, this may improve the evidence base for management of this substantial but understudied population Table 1.

## Summary

In this Masterclass, we described the prevalence of multimorbidity in the context of CLBP and the negative impacts this has on outcomes for these individuals. We presented data outlining why physical activity and exercise are key components of a multimodal lifestyle intervention to address this problem, and highlighted important considerations for clinicians to help their clients with CLBP and other long-term health conditions become more physically active. Potential barriers to this approach, particularly around the scope of practice and clinical responsibility, were also discussed.

**Funding:** This research did not receive any specific grant from funding agencies in the public, commercial, or not-for-profit sectors.

## Declarations of competing interest

MDJ has received research grant funding from the National Health and Medical Research Council of Australia. All funds were paid to his institution. MDJ has also received sittings fees for his role on committees for Exercise and Sports Science Australia. MLF has received research grant funding from the National Health and Medical Research Council of Australia and multiple grants from other national and international research foundations. All funds were paid to her institution. MLF has also provided expert opinion to the advisory board of VIATRIS Medical Affairs and received payment for this role.
